# Probing, Stuffing and Syringing the Ear

**Published:** 1869-04

**Authors:** 


### Probing, Stuffing and Syringing the Ear.

The practice so prevalent of probing the ear is one of very serious injury. Many persons carry in their pockets a pair of little tweezers, upon the end of which is an "ear-spoon," a villianous little instrument, productive of much harm. With this it is the custom to probe and prick at the ears, striving to remove the cerumen which nature intended should remain where it is placed.

The physician who is accustomed to diseases of the ear, rarely does any probing. If instrumental interference is necessary he approaches it with the utmost caution, as no part of the anatomy is more sensative than the lining membrane of the ear-passage. By rubbing or poking at the ears with the view of relieving itching, an inflammation is instituted which hardens the cerumen and renders the passage dry and irritable.

Squirting water into the ears with those little glass syringes is another bad practice, for the reason that those who are unfamiliar with the anatomy of the ear, are continually liable either to poke the drum with the nozzle of the syringe, or to use too much force in injecting the fluid. To syringe the ear properly and effectually is by no means a simple operation, and our experience teaches us that very many persons have been rendered deaf entirely, from unskillful and useless syringing.

The practice of introducing laudanum, onion-juice, sweet oil and the like, in the ear, is another very common and hurtful proceeding. *Oils* of any kind should never be used in the ear.—They soon become rancid and gummy, adhering to the drum and causing dullness of hearing, while laudanum dropped into the ear will scald and irritate both the lining membrane and drum. The best application that can be used at home, for ear-ache, is equal parts of pure glycerine and laudanum, well shaken together, and warmed by placing the bottle in hot water. A few drops of this placed in the ear and confined there with cotton-wool, will generally give relief.

Foreign bodies are apt to get into the ears of children. Beans, peas, buttons, corn, seeds, pebbles, bits of slate pencils, &c., are often thrust into the ears by young experimenters. In such cases it is better to apply at once to a competent surgeon, and upon no account should an instrument be introduced into the ear without the most careful examination, else the offending particle might be thrust against or even through the drum and so permanently destroy the hearing. A physician skilled in the management of the ear can remove the difficulty by means of a careful system of syringing. Many persons regard the ear as a mere hole, and squirt away at it, much upon the same principle that a boy would adopt in drowning out a rat.

